# Imaging PARP with [^18^F]rucaparib in pancreatic cancer models

**DOI:** 10.1007/s00259-022-05835-4

**Published:** 2022-05-26

**Authors:** Chung Ying Chan, Zijun Chen, Gianluca Destro, Mathew Veal, Doreen Lau, Edward O’Neill, Gemma Dias, Michael Mosley, Veerle Kersemans, Florian Guibbal, Véronique Gouverneur, Bart Cornelissen

**Affiliations:** 1 Oxford Institute for Radiation Oncology, Department of Oncology, University of Oxford, Old Road Campus Research Building, Off Roosevelt Drive, OX3 7DQ Oxford, UK; 2 Department of Chemistry, Chemistry Research Laboratory, University of Oxford, 12 Mansfield Road, Oxford, OX1 3TA UK; 3 Department of Nuclear Medicine and Molecular Imaging, University Medical Center Groningen, University of Groningen, Groningen, The Netherlands

**Keywords:** PARP, Rucaparib, [^18^F]Rucaparib, PET imaging, Pancreatic cancer

## Abstract

**Purpose:**

Rucaparib, an FDA-approved PARP inhibitor, is used as a single agent in maintenance therapy to provide promising treatment efficacy with an acceptable safety profile in various types of *BRCA*-mutated cancers. However, not all patients receive the same benefit from rucaparib-maintenance therapy. A predictive biomarker to help with patient selection for rucaparib treatment and predict clinical benefit is therefore warranted. With this aim, we developed [^18^F]rucaparib, an ^18^F-labelled isotopologue of rucaparib, and employed it as a PARP-targeting agent for cancer imaging with PET. Here, we report the *in vitro* and *in vivo* evaluation of [^18^F]rucaparib in human pancreatic cancer models.

**Method:**

We incorporated the positron-emitting ^18^F isotope into rucaparib, enabling its use as a PET imaging agent. [^18^F]rucaparib binds to the DNA damage repair enzyme, PARP, allowing direct visualisation and measurement of PARP in cancerous models before and after PARP inhibition or other genotoxic cancer therapies, providing critical information for cancer diagnosis and therapy. Proof-of-concept evaluations were determined in pancreatic cancer models.

**Results:**

Uptake of [^18^F]rucaparib was found to be mainly dependent on PARP1 expression. Induction of DNA damage increased PARP expression, thereby increasing uptake of [^18^F]rucaparib. *In vivo* studies revealed relatively fast blood clearance of [^18^F]rucaparib in PSN1 tumour-bearing mice, with a tumour uptake of 5.5 ± 0.5%ID/g (1 h after i.v. administration). *In vitro* and *in vivo* studies showed significant reduction of [^18^F]rucaparib uptake by addition of different PARP inhibitors, indicating PARP-selective binding.

**Conclusion:**

Taken together, we demonstrate the potential of [^18^F]rucaparib as a non-invasive PARP-targeting imaging agent for pancreatic cancers.

**Supplementary Information:**

The online version contains supplementary material available at 10.1007/s00259-022-05835-4.

## Introduction

Pancreatic ductal adenocarcinoma (PDAC) is a highly fatal human malignancy with poor prognosis [1]. Incidence of pancreatic cancer is increasing globally for men and women with 5-year survival rate <10% [2, 3]. Due to the deep-seated location of the pancreas and the asymptomatic nature of PDAC at earlier stages of the disease, most patients present with advanced lesions at the time of diagnosis [4]. Surgical resection is considered the most effective treatment for pancreatic cancer; however, only 20% of patients are eligible for initial resection, with >90% of patients experiencing relapse and eventually succumbing to the disease [5]. Chemotherapy is the main treatment for PDAC. However, resistance is common, leading to poor treatment outcomes. The various molecular subgroups of PDAC with unique biological characteristics make it even tougher to treat with the right chemo- or chemo-radiotherapy regime [3]. Novel treatments and accompanying selection biomarkers are therefore needed.

Inhibition of poly(ADR-ribose) polymerase (PARP) as a cancer therapy is a recent, successful strategy for various cancer treatments, including pancreatic cancer, based on the concept of synthetic lethality. PARP inhibitors impair the processing of single-strand break (SSB) repair by inhibiting the catalytic activity of the DNA damage repair enzyme, PARP, and by binding and trapping the PARP enzyme onto broken DNA, thereby causing stalling and collapse of replication forks or by accelerating fork elongation, leading to double-strand break (DSB) formation. These DSBs require homologous recombination (HR) for repair during S phase [6]. Therefore, in cancer cells with mutations in HR genes, such as *BRCA1*/2, DSBs cannot be repaired, causing cell death. Currently, five PARP inhibitors (olaparib, rucaparib, niraparib, talazoparib, veliparib) have been approved for use in a variety of cancers with HR mutations, and it is known that each of the inhibitors possesses different target-binding profiles as demonstrated in the *in vitro* assays [7, 8]. To date, only olaparib has been approved as maintenance therapy for patients with *gBRCA1/2* pathogenic variants and platinum-sensitive, metastatic PDAC [9, 10]. Recently, rucaparib showed promising efficacy with an acceptable safety profile for patients with advanced pancreatic cancer [11]. Another phase II study showed that two-thirds of pancreatic cancer patients with *BRCA1/2* and *PALB2* variants benefited from rucaparib treatment [12]. Despite promising outcomes using PARP inhibitor therapy in patients with various types of cancer, selecting those patients who will benefit from this therapy remains one of the major challenges, since heterogeneous responses to PARP inhibitor therapy are often observed even in patients preselected for *BRCA*-mutated cancers, which may be associated with complex intrinsic or acquired resistance [13]. Genetic testing using, for example, BRACAnalysis® or myChoice® (Myriad Genetics), has been implemented in many clinical trials to determine HR deficiency status and aid with patient selection for PARP inhibition therapy. Nonetheless, multiple reports have shown that the presence of a *BRCA* mutation does not always result in synthetic lethality with PARP inhibitor therapy in cancer [6], indicating that HR deficiency may not be the only predictive factor for PARP inhibitor treatment. It has also been shown that in cancers that respond to PARP inhibitor therapy, those cancers expressing more PARP enzyme are more sensitive. Combining genetic testing and measurement of PARP expression in tumour may increase the accuracy in patient stratification and prediction of therapy efficacy. Therefore, the development of imaging tools to visualise and quantify PARP expression is warranted.

Molecular imaging using radiolabelled PARP-targeting agents can serve as a powerful tool for non-invasive *in vivo* PARP imaging [6, 14]. Incorporating a radionuclide into a PARP inhibitor allows PET (^11^C, ^18^F) or SPECT (^123^I) imaging for *in vivo* visualisation of PARP expression, PARP inhibitor distribution, drug-target engagement and tumour uptake, thus providing important clinical information for diagnosis and staging, selection of patient subgroups suitable for PARP inhibition therapy, monitoring treatment response to genotoxic treatments or gauging the emergence of resistance. Numerous radiolabelled PARP-targeting agents have been developed and described in recent reviews [6, 14]. Among them, [^18^F]olaparib [15] and [^18^F]talazoparib [16, 17] are ^18^F-labelled radioisotopologues, i.e. they have chemical structures identical to their parent molecules; hence, they display identical pharmacokinetic, pharmacodynamic and binding specificity profiles *in vivo*. Therefore, these ^18^F-labelled radioisotopologues can be used to accurately determine drug distribution and measure drug-target engagement as their non-labelled compounds, and provide crucial bio-information for drug dosimetry calculation and monitoring treatment response for cancer patients undergoing particular type of PARP inhibitor treatment. Recently, we have developed an ^18^F-labelled isotopologue of rucaparib, [^18^F]rucaparib [18], which could serve as a PARP-targeting PET imaging tool and provide useful clinical information in PARP- or chemo- therapies for cancer. In this study, we report the *in vitro* and *in vivo* evaluation of [^18^F]rucaparib in PDAC models and demonstrate its potential as a non-invasive PARP imaging agent.

## Materials and methods

### General

Unless otherwise indicated, all reagents were purchased from Sigma–Aldrich and used without further purification. Olaparib, rucaparib, veliparib, talazoparib and niraparib were purchased from Stratech Scientific Ltd. (UK). Daidzin and mycophenolate mofetil were purchased from Cambridge Bioscience Ltd. (UK) and Insight Biotechnology Ltd, respectively. [^18^F]olaparib and [^18^F]rucaparib were synthesised as described [15, 18].

### Cell culture

Pancreatic ductal adenocarcinoma cells: AsPC1 and PSN1, were purchased from ATCC (UK) and maintained in Roswell Park Memorial Institute (RPMI) supplemented with 10% foetal bovine serum (FBS, Gibco), 2 mM L-glutamine, 100 units/mL penicillin, and 0.1 mg/mL streptomycin (Gibco). Cells were grown in a humidified environment at 37 °C and 5% CO_2_. Cells were harvested and passaged using trypsin–EDTA solution. Cells were used no more than 25 passages following resuscitation from liquid nitrogen storage. Cells were authenticated by STR profiling and tested regularly for the absence of mycoplasma contamination. RIPA buffer (950 mM Tris pH 8.0, 1% NP40, 0.5% sodium deoxycholate, 0.1% sodium dodecyl sulphate and 150 mM sodium chloride) was used for cell lysis.

### Cellular protein expression in PDAC cells

Relative expression of PARP1, 2 and 3, ALDH2 and IMPDH2 was determined by flow cytometry. Cells (1 × 10^6^ cells/well) were seeded in 96-well plates in growth medium, washed with FACS buffer (PBS, 2% FBS, 1 mM EDTA, 0.1% NaN_3_) and centrifuged at 500×g for 5 min. Immunostaining was performed using the Foxp3/transcription factor staining buffer set (eBioscience^TM^, USA). Intracellular staining was conducted in permeabilisation buffer for 30 min at 4 °C in the dark using the following antibodies: AF488-conjugated anti-PARP-1 (1:100; sc-80070), AF594-conjugated anti-PARP-2 (1:100; sc-393310), anti-PARP-3 (1:100; sc-390771) or anti-PARP-tankyrase-1/2 (1:100; sc-365897) from Santa Cruz Biotechnology Inc. (USA), AF488-conjugated anti-IMPDH2 (1:500; ab-200770) from Abcam plc. (UK) and AF488-conjugated anti-ALDH2 (1:100, ABIN6817568) from Antibodies-online GmbH (UK). Fixable viability dye ef780 (1:4000; eBioscience^TM^; 65-0865-14) was used for live and dead cells discrimination. Fixation of immunostained cells was performed for 15 min at room temperature. Flow cytometry was conducted on the CytoFLEX benchtop flow cytometer (Beckman Coulter, USA), with appropriate lasers and filters, positive and negative controls. Data were analysed using FlowJo^TM^ (Tree Star Inc., BD Biosciences, USA).

### *In vitro* uptake and binding selectivity of [^18^F]rucaparib

AsPC1 (1 × 10^5^ cells/well) and PSN1 cells (7.5 × 10^4^ cells/well) were seeded separately in 24-well plates (in 300 μL growth medium) and allowed to adhere for at least 20 h. Cells were washed and exposed to [^18^F]rucaparib ([^Total^F]rucaparib: 64 nM [3 GBq/μmol] and 258 nM [4.8 GBq/μmol], separately) and the cells incubated at 37 °C for 30 min. To assess [^18^F]rucaparib-binding selectivity, unlabelled PARP inhibitors, IMPDH2 or ALDH2 inhibitor, were added (100 μM, in 270 μL growth medium) for 45 min at 37 °C before addition of [^18^F]rucaparib (156 kBq, 6.8 GBq/μmol; [^Total^F]rucaparib final concentration: 100 nM), and the cells were incubated at 37 °C for a further 45 min. Cell culture medium was removed, and cells were washed with PBS. Cells were lysed using RIPA buffer for 15 min at room temperature, and the amount of ^18^F in the cell lysates was measured using an automated gamma counter (PerkinElmer), normalising for the number of cells.

To determine the time dependence uptake of [^18^F]rucaparib, cells were seeded separately in 24-well plates as above and exposed to [^18^F]rucaparib (100 kBq, 4.8 GBq/μmol; [^Total^F]rucaparib final concentration: 258 nM) at 37 °C for different intervals (1–120 min). Cells were lysed and the amount of ^18^F in the cell lysates was measured as above.

### Cell retention of [^18^F]rucaparib and [^18^F]olaparib

AsPC1 (1 × 10^5^ cells/well) and PSN1 cells (7.5 × 10^4^ cells/well) were exposed to [^18^F]rucaparib (100 kBq, 4.8 GBq/μmol; [^Total^F]rucaparib final concentration: 258 nM) or [^18^F]olaparib (400 kBq, 2.8 GBq/μmol: [^Total^F]olaparib final concentration: 242 nM) in 24-well plates at 37 °C for 30 min. Cell culture medium was removed, and cells were washed with PBS, followed by the addition of fresh medium (300 μL). Cells were then further incubated at 37 °C. Cell culture medium was removed, and cells were washed with PBS at different times (0–3 h). Cells were lysed using RIPA buffer for 15 min at room temperature, and the amount of ^18^F in the cell lysates was measured as above.

### Uptake and retention of [^18^F]rucaparib and [^18^F]olaparib in cells treated with DNA-damaging reagents

AsPC1 (1 × 10^5^ cells/well) and PSN1 cells (7.5 × 10^4^ cells/well) were exposed to methyl methanesulfonate (MMS) or temozolomide (TMZ) (100 μM, in a total of 270 μL growth medium) for 3 h at 37 °C. Additionally, [^18^F]rucaparib (500 kBq, 3.6 GBq/μmol; [^Total^F]rucaparib final concentration: 301 nM) or [^18^F]olaparib (400 kBq, 8.5 GBq/μmol; [^Total^F]olaparib final concentration: 300 nM) was added, and the cells were incubated at 37 °C for 30 min. Cell culture medium was removed, and cells were washed with PBS. For cell retention, fresh medium (300 μL) was added to the cells. Cells were then further incubated at 37°C for 3 h. Cell culture medium was removed, and the amount of ^18^F in the cell lysates was measured as above. Protein expression of the cells after DNA-damaging treatments (or PARP inhibitors 10 μM) was performed using flow cytometry as above.

### PET/CT imaging and biodistribution of [^18^F]rucaparib

Female Balb/c nu/nu (OlaHsd-*Foxn1*^*nu*^) mice, aged 4–6 weeks, were purchased from Envigo (UK). Animals were housed in IVC cages, up to 6 per cage, in an artificial day–night cycle facility. Food and water were provided *ad libitum*.

PSN1 cells were harvested using trypsin, washed twice with PBS and reconstituted in PBS:Matrigel® Matrix High Concentration (1:1). Cell suspensions (PSN1: 2 × 10^6^ cells/ 100 μL) were injected subcutaneously in the lower right flank.

Animals were administered [^18^F]rucaparib (0.87–11.38 MBq in 100 μL of PBS, A_m_ = 1.5–30.9 GBq/μmol) by intravenous injection via the lateral tail vein. To evaluate the selectivity of tumour uptake, an excess of unlabelled rucaparib or olaparib (0.5 mg) was co-administered as a blocking agent in some animals. Dynamic PET images (1 h) were acquired using a MILabs VECTor^4^ camera, equipped with an ultra-high resolution rat/mouse collimator (1.8 mm), followed by a cone-beam CT scan (55 kV, 0.19 mA) for anatomical reference and attenuation correction. Animal was anesthetised by 4% isoflurane gas (0.5 L/min O_2_) and maintained at 2.5% at 37 °C throughout the duration of image acquisition. PET images were reconstructed using U-SPECT-Rec3.22 software (MILabs, Utrecht, The Netherlands), applying a pixel-based algorithm, ordered subset expectation maximisation (OSEM) with 6 subsets, 4 iterations and 0.8 mm voxel size for fluorine-18 (energy window settings 477.9–584.1 keV). Reconstructed PET and CT images were viewed and analysed using PMOD v.3.37 (PMOD Technologies, Zurich, Switzerland).

Either 1 h or 2 h after radiolabelled compound injection, animals were euthanised. Selected organs, tissues and blood were removed, and the percentage of the injected dose per gram of tissue (%ID/g) was determined, using a HIDEX automated gamma counter.

*Ex vivo* localisation of [^18^F]rucaparib in PSN1 xenografts was determined using autoradiography of tumour sections (10 μm). Uptake in the same tumour sections was compared to immunohistochemistry staining for PARP1, 2 and 3. Full details of procedures and protocols are provided in Supplemental Information.

### Statistical analysis

All data were obtained at least in triplicate. All statistical analyses and nonlinear regression were performed using GraphPad Prism v6 (GraphPad Software, San Diego, CA, USA). Data were tested for normality and analysed as appropriate by one-way ANOVA. Results are reported as mean ± SD, unless stated otherwise.

## Results

### Protein expression levels in AsPC1 and PSN1 cells

PARP inhibitors, including rucaparib, interact with PARP enzymes (PARP1, 2, 3, tankyrase1/2) as well as with other enzymes such as ALDH2 and IMPDH2 [8]. The relative expression levels of these enzymes in AsPC1 and PSN1 cells were measured using flow cytometry (Supplemental Figure S1). Expression of PARP1 was higher in PSN1 cells than in AsPC1 cells, which is consistent with previously reported [19].

### [^18^F]rucaparib uptake and its binding selectivity in AsPC1 and PSN1 cells

**Fig. 1:**
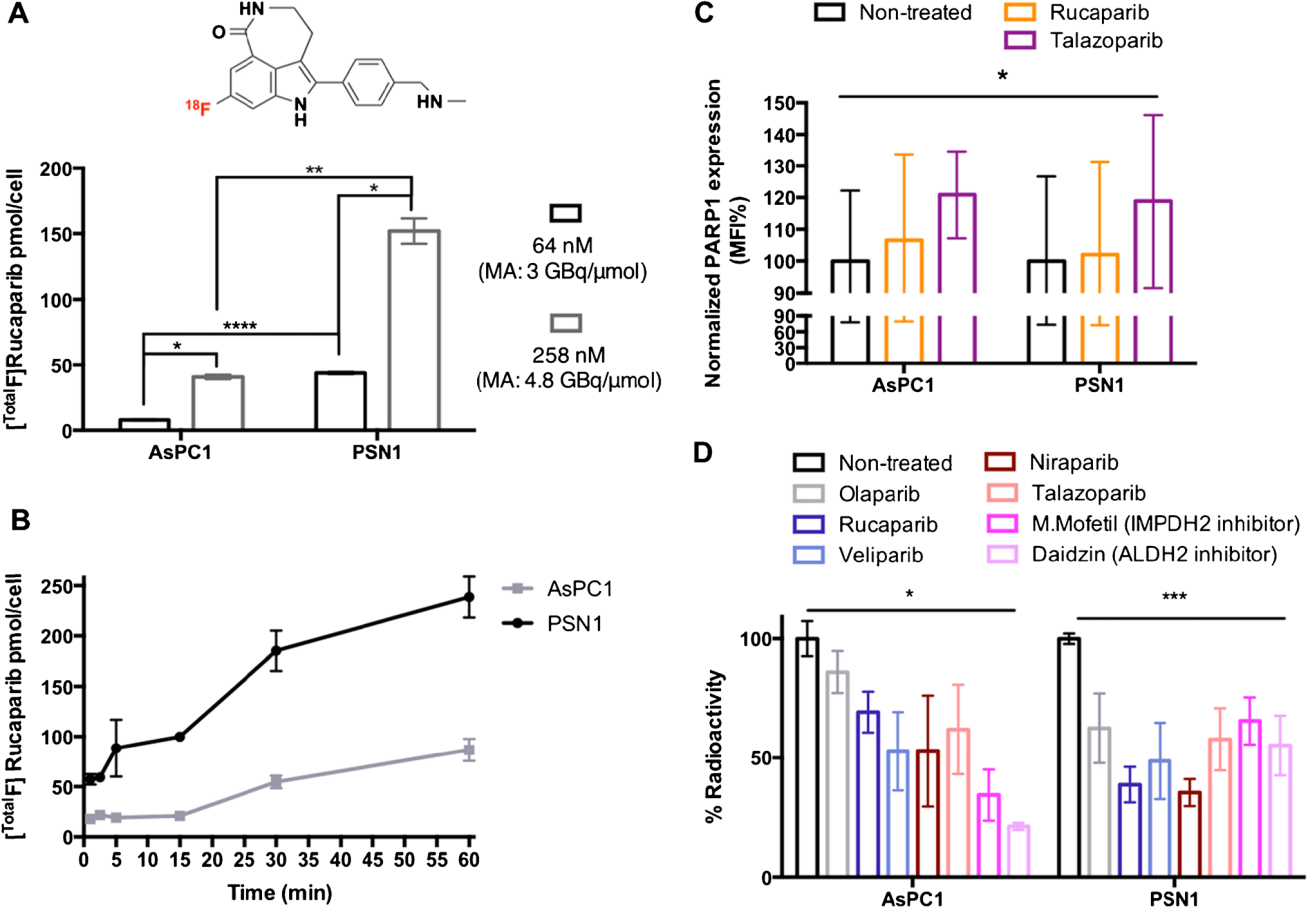
**A** Chemical structure of [^18^F]rucaparib and its uptake in AsPC1 and PSN1 cells at different concentrations of [^Total^F]rucaparib. **B** Time dependency uptake assay of [^Total^F]rucaparib ([^18^F]rucaparib: 100 kBq, 4.8 GBq/μmol) in AsPC1 and PSN1. **C** Mean fluorescence intensity (MFI) of PARP1 staining in AsPC1 and PSN1 cells after exposure of PARP inhibitors (10 μM) for 3 h, assessed by flow cytometry analysis. **D** Blocking of [^18^F]rucaparib (156 kBq, 6.8 GBq/μmol) uptake in AsPC1 and PSN1 cells with one of a panel of unlabelled PARP inhibitors, ALDH2 inhibitor and IMPDH2 inhibitor (100 μM). Asterisks indicate levels of significance: *, *P* < 0.05; **, *P* < 0.01; ***, *P* < 0.001; and ****, *P* < 0.0001

The uptake of [^18^F]rucaparib was evaluated in AsPC1 and PSN1 cells based on the amount of ^18^F. Cell association of [^18^F]rucaparib was higher in PSN1 cells than in AsPC1 cells (Fig. 1A), correlating with PARP1 expression of these cells. Cellular uptake was relatively fast (<1 min, Fig. 1B). Increased uptake was observed when higher amounts of [^Total^F]rucaparib were presented to the cells (Fig. 1A) or the cells were exposed to [^Total^F]rucaparib for longer periods of time (Fig. 1B). PARP1 enzyme was also upregulated after exposure of PARP inhibitors (such as rucaparib and talazoparib, 10 μM, Fig. 1C). These suggest that treatment of PARP inhibitors may alter or upregulate DNA damage repair proteins, such as PARP1, thereby increasing [^18^F]rucaparib uptake. Further investigation is needed to gain better understanding on PARP inhibition mechanism.

Binding selectivity of [^18^F]rucaparib was evaluated via blocking with structurally different PARP inhibitors and inhibitors of other NAD^+^-dependent enzymes, such as ALDH2 and IMPH2 (daidzin and mycophenolate mofetil, respectively), which were previously identified as a binding target of PARP inhibitors [20, 21]. The addition of excess unlabelled PARP, ALDH2 and IMPDH2 inhibitors (100 μM) was able to significantly reduce the uptake of [^18^F]rucaparib in both AsPC1 and PSN1 cells (Fig. 1D), indicating substantial binding of [^18^F]rucaparib to these enzymes. The PARP inhibitors were able to reduce [^18^F]rucaparib uptake to a greater extent in PSN1 than in AsPC1 cells. This may be due to the higher PARP1 expression level in PSN1 cells. The ALDH2 inhibitor could significantly block the uptake of [^18^F]rucaparib in both AsPC1 and PSN1 cells, suggesting that [^18^F]rucaparib may possibly internalise in mitochondria and bind to ALDH2 or that this system lowers [18F]rucaparib uptake by another, as yet unelucidated mechanism.

### DNA-damaging treatments upregulate protein expression, including PARP, and increase uptake and retention of PARP inhibitors in cells

**Fig. 2:**
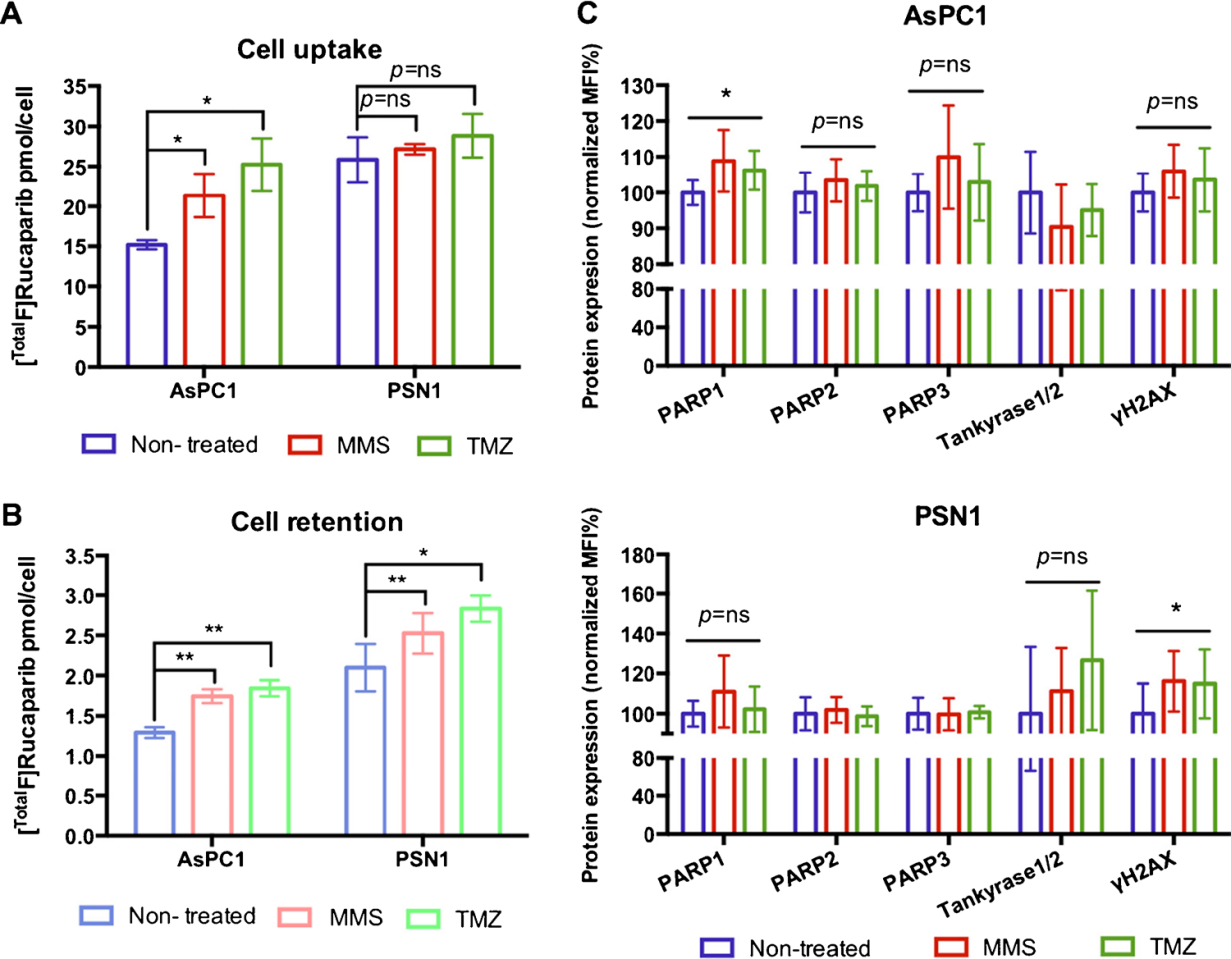
**A** Uptake and **B** retention (at 3 h) of [^Total^F]rucaparib ([^18^F]rucaparib: 500 kBq, 3.6 GBq/μmol) in cells (AsPC1 and PSN1) treated with DNA-damaging reagents (MMS and TMZ). **C** Mean fluorescence intensity (MFI) for PARP1, 2 and 3, tankyrase1/2 and γ-H2AX expressions in AsPC1 and PSN1 cells after exposure of MMS or TMZ (100 μM) for 3 h, assessed by flow cytometry analysis. Asterisks indicate levels of significance: ns, *P* > 0.05; *, *P* < 0.05; **, *P* < 0.01

Since rucaparib and olaparib are known to display different binding properties towards PARP and potentially other proteins [7, 8], cellular uptake and retention of both PARP inhibitors were compared using their ^18^F-labelled isotopologues in AsPC1 and PSN1 cells. The results showed that far higher (100-fold) [^Total^F]rucaparib uptake and retention were observed in both cell lines, compared with [^Total^F]olaparib (Fig. 2A and B; Supplemental Figures S3–4). The amount of either PARP inhibitor retained in cells at 3 h after removal also correlated with the PARP1 expression levels, with PSN1 cells possessing higher PARP expression levels and retaining more rucaparib and olaparib. Efflux of both rucaparib and olaparib was biphasic with relatively fast exchange, with more than 50% exiting cells in the first 10–30 min, followed by slow efflux over the next 2.5 h (Supplemental Figure S4), in concordance with the cellular elimination pattern reported for [14C]rucaparib [22]. However, the weighted cellular efflux half-life of rucaparib was found 49.9 ± 6.8 min and 43.9 ± 3.2 min in AsPC1 and PSN1 cells, respectively, which is considerably slower than the previously reported half-life of 20 min in SW620 cells [22].

MMS and TMZ are DNA alkylating agents that cause SSBs, which may eventually convert to DSBs [23, 24]. These events trigger DNA damage signalling, thereby causing recruitment or upregulation of DNA damage repair proteins, e.g. PARP. Exposure to these DNA-damaging reagents increased cell association and retention of both [^Total^F]rucaparib and [^Total^F]olaparib in cells (Fig. 2A and B; Supplemental Figure S3). MMS or TMZ induced DNA damage, as indicated by the increasing trend of γH2AX expression in the treated cells (Fig. 2C) although this observation is not statistically significant in AsPC1 cells. MMS and TMZ also triggered upregulation of PARP proteins (<10%), but not IMPDH2, in both cell lines (Fig. 2C; Supplemental Figures S5–6). This observation may indicate that DNA breaks induced upregulation of PARP enzymes, which may in turn recruit for the binding of radiolabelled PARP inhibitor. Although the increased uptake and retention of the PARP inhibitors may be attributed by the upregulation of PARPs enzymes, upregulation of other off-target proteins, such as ALDH2 (as shown in Supplemental Figures S5–6) may also contribute.

### Pharmacodynamics of [^18^F]rucaparib in PSN1 tumour-bearing mice

**Fig. 3:**
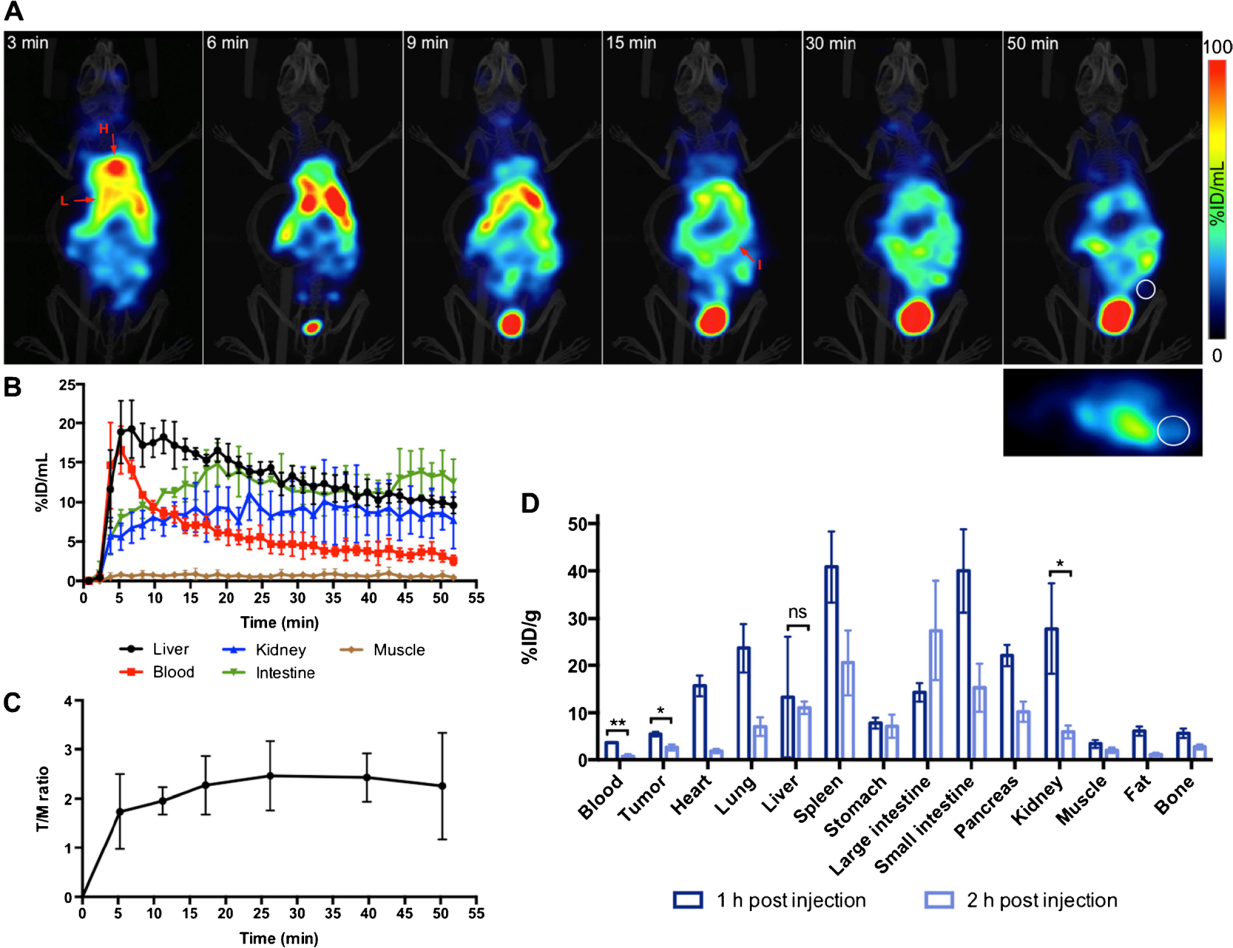
**A** Representative dynamic PET images after intravenous injection of [^18^F]rucaparib (2.38 MBq, 30.9 GBq/μmol). Middle of time frames is indicated in minutes. Images are presented as maximum intensity projections. H = Heart; I = Intestine; L = Liver; White circle = Tumour. **B** Time activity curves based on VOI analysis of dynamic images of PSN1 xenografts (n=3). **C** Tumour-to-muscle (T/M) ratio of [^18^F]rucaparib over time. **D** Biodistribution of [^18^F]rucaparib in selected tissues in PSN1 tumour-bearing mice, 1 or 2 h after intravenous administration of [^18^F]rucaparib (0.87–11.38 MBq, 1.5–30.9 GBq/μmol). Asterisks indicate levels of significance: ns, *P* > 0.05; *, *P* < 0.05; **, *P* < 0.01

Dynamic imaging (Fig. 3A−C) together with biodistribution (Fig. 3D) following administration of [^18^F]rucaparib (2.38–11.38 MBq, 30.9 GBq/μmol) to PSN1 tumour-bearing mice (n=3) showed that [^18^F]rucaparib is eliminated via multiple pathways, such as hepatobiliary and renal, resulting in uptake in liver, small and larger intestines, kidneys and bladder. The clearance pattern from the biodistribution data of [^18^F]rucaparib is similar to the clearance pattern of [^18^F]olaparib in the same xenograft mice [15], or [^18^F]FTT in naïve mice [25] with high uptake in organs such as liver, small and larger intestines and kidneys, where [^18^F]FTT is a ^18^F-labelled rucaparib-like compound, the subject of a number of clinical trials [26, 27]. Volume-of-interest analysis of the ^18^F signal (Fig. 3B, C) demonstrated fast tumour uptake of [^18^F]rucaparib (within 10 min), followed by relatively slow clearance. Blood clearance of [^18^F]rucaparib followed a similar biphasic pattern as [^18^F]olaparib in the same xenograft mice, with fast and slow half-life of 1.7 ± 0.44 min (54 ± 10%; 95%CI, 0.77–4.6 min) and 16 ± 10 min (46 ± 10%; 95%CI, 6.1–37 min), respectively, resulting in a weighted half-life of 4.3 ± 0.62 min, which is shorter than the weight blood half-life of [^18^F]olaparib (32.3 min) [15]. The half-life for liver elimination of [^18^F]rucaparib was 31 ± 19 min. Uptake in kidney and bladder (within 5 min, Fig. 3A, B) also indicates fast renal clearance of [^18^F]rucaparib. Given the very low injected dose of the compound (8.9 μg or 27.5 nmol/kg), and the intravenous administration route, the blood half-life of [^18^F]rucaparib measured here was markedly shorter than that previously reported, although this was using other, inherently slower administration routes (1.4–1.5 h; after 10 mg/kg, administered orally [28] or intraperitoneally [29]).

**Fig. 4:**
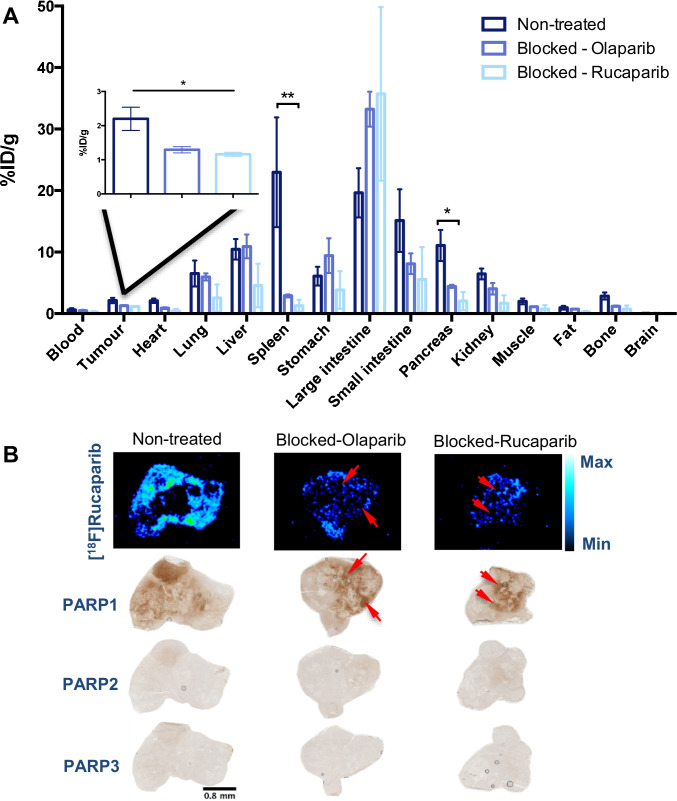
**A** Biodistribution in selected tissues in PSN1 tumour-bearing mice 2 h after intravenous injection of [^18^F]rucaparib (0.87–2.47 MBq, A_m_ = 5.5 GBq/μmol) (n=3/group), with or without an excess of unlabelled olaparib or rucaparib (0.5 mg). **B** Autoradiography of PSN1 tumour sections showing ^18^F localisation and the PARP1 occupancy by unlabelled PARP inhibitors (olaparib and rucaparib, red arrows), and immunohistochemical staining of PSN1 tumour sections showing PARP1, 2 and 3 expression. Asterisks indicate levels of significance: *, *P* < 0.05; **, *P* < 0.01

Tumour uptake of [^18^F]rucaparib in PSN1 xenografts was measured to be 5.49 ± 0.49%ID/g and 2.2 ± 0.34%ID/g at 1 and 2 h post-administration, respectively, with tumour-to-blood (T/B) ratios of 1.53 ± 0.11 and 4.33 ± 2.1, suggesting relatively slow tumour clearance compared with blood and liver clearances. Tumour uptake of [^18^F]rucaparib was markedly higher than that of [^18^F]olaparib (3.16 ± 0.36%ID/g; *P* < 0.01) in the same PSN1 xenografts at 1 h post-injection reported previously [15]. The tumour-to-muscle (T/M) ratio of [^18^F]rucaparib from PET/CT images (2.3 ± 1.1 at 50 min post-injection, Fig. 3C) is comparable to the ratio of [^18^F]FTT in breast cancer xenografts (1.2–1.9 at 1 h post-injection [25, 30]), showing the good tumour-to-background ratio and potentials of [^18^F]rucaparib as imaging agent. [^18^F]Rucaparib uptake in PSN1 tumour-bearing mice was reduced significantly by co-administration of a therapeutic dose of unlabelled PARP inhibitors rucaparib or olaparib (2.2 ± 0.34%ID/g to 1.16 ± 0.06%ID/g or 1.3 ± 0.09%ID/g, respectively; *P* < 0.05), indicating PARP-selective uptake *in vivo* (Fig. 4A). Similar blocking effects were observed in PARP-expressing organs, such as spleen and pancreas, further supporting the binding selectivity of [^18^F]rucaparib towards PARPs. Furthermore, *ex vivo* analysis combining immunohistochemical staining and autoradiography of PSN1 tumour tissues (Fig. 4B) demonstrated that the uptake of [^18^F]rucaparib was correlated with PARP expression, mainly PARP1. PARP1 expression in PSN1 tumours was heterogeneous, hence resulting in heterogeneous uptake of [^18^F]rucaparib. The ^18^F signals were significantly reduced in the sections from tumours treated with either olaparib or rucaparib, demonstrating unlabelled drug occupancy in the tumour sections (red arrows, Fig. 4B).

## Discussion

Rucaparib (Rubraca) is a potent PARP inhibitor and approved as a monotherapy treatment for patients with *BRCA*-mutated (germline and/or somatic), platinum-sensitive or relapsed ovarian cancer and castration-resistant prostate cancer [31]. Additionally, its clinical application has been recently expanded to advanced pancreatic cancer (*BRCA* mutated or platinum sensitive) [11, 12], which is well known as one of the tough-to-treat cancers, with promising efficacy although some of the patients remained irresponsive to rucaparib treatment. The development of an ^18^F-labelled version of rucaparib may help in overcoming the challenge of patient stratification for rucaparib treatment by visualisation of PARP expression levels in patients and allow determining drug distribution, measuring drug engagement and monitoring treatment response. The aim of this study was to evaluate the potential of [^18^F]rucaparib, as a PARP-targeting PET agent, in human pancreatic cancer models *in vitro* and *in vivo*.

*In vitro* and *in vivo* studies (uptake, retention and selectivity) of [^18^F]rucaparib in AsPC1 and PSN1 models, which display different PARP expression levels, suggested that PARP1 remains one of the key players responsible for [^18^F]rucaparib uptake in cells, although [^18^F]rucaparib has binding selectivity towards other proteins, leading to higher cellular and tumour uptake compared with the uptake of [^18^F]olaparib in the same models. Our studies showed that rucaparib could interact with other biomolecules or cancer-related proteins present in different organelles, which compose a catalytic domain with a NAD^+^ binding site, such as ALDH2. Hence, further investigations on identifying rucaparib-binding targets, including binding affinity, protein and inhibitor trapping, etc., are necessary to understand the full mechanism of PARP inhibitor treatment and imaging with radiolabelled PARP inhibitors. We hypothesised that the binding selectivity profile of rucaparib and the previously identified better retention in cells [8, 22] should result in higher tumour uptake and retention, and better contrast versus normal tissues of [^18^F]rucaparib in cancer cells, providing good tumour-to-background ratio and making localisation of tumour more straight forward. An in-depth insight of the effect of molar activity and administered amounts on [^18^F]rucaparib uptake in PARP-expressing cancers versus normal tissue will be investigated and reported in due course.

Over the past 15 years, numerous radiolabelled PARP inhibitors have been developed as PARP-targeting imaging agents or radionuclide therapy agents [6]. Despite good PARP selectivity, all these agents, including [^18^F]rucaparib described here, share hepatobiliary clearance patterns with high uptake in abdominal organs. Some of these imaging agents, such as [^18^F]PARPi-FL [32], [^124/131^I]PARPi [33] and [^125^I]KX2 [34], also suffer from relatively low tumour uptake (<1%ID/g). These may make them less favourable for imaging abdominal cancers, such as pancreatic cancers. On the other hand, [^18^F]rucaparib displays a more favourable *in vivo* pharmacokinetics, such as relatively fast and high tumour uptake with longer tumour retention, and potentially can serve as a better PARP imaging agent for visualisation of PARP expression levels in cancers. However, more studies are warranted to investigate the utility of [^18^F]rucaparib in imaging of abdominal cancer such as pancreatic cancers. For example, an optimised imaging dose and time to improve tumour delineation, or the use of appropriate orthotopic or patient-derived xenografts would help to address the suitability of [^18^F]rucaparib for imaging of pancreatic cancer in clinical practice. Although further in-depth understanding of [^18^F]rucaparib metabolism remains warranted, [^18^F]rucaparib is an isotopologue of the FDA-/EMA-approved drug rucaparib, which benefits from a wealth of clinical data and toxicity data already available that will aid its translation to the clinic [35].

Previously, several studies demonstrated the use of radiolabelled modified PARP inhibitors to probe PARP and PARP inhibitor distribution and their target engagement [36, 37]. For example, a fluorescence-labelled PARP inhibitor, PARP-FL, was used to predict drug distribution and target engagement of rucaparib at an intracellular level [36], while [^18^F]FTT can visualise the pharmacodynamics of PARP inhibitors *in vivo* [37]. Given the comparable tumour-to-background ratio as [^18^F]FTT in preclinical PET imaging, [^18^F]rucaparib, as an exact chemical match of rucaparib together with the fluorescence properties of rucaparib (absorption and emission at excitation wavelengths 355 and 405 nm [36]), conveniently would allow direct imaging of the delivery and engagement of rucaparib (or even other PARP inhibitors) in intracellular or in tumour tissue using various imaging techniques (PET, fluorescence microscope and flow cytometry). Our *in vivo* and *ex vivo* studies demonstrated the potential of [^18^F]rucaparib as a direct companion imaging agent, showing the pharmacodynamics and target engagement of rucaparib, which will offer a variety of clinically relevant applications, such as patient selection and treatment prediction.

Chemotherapy is one of the most common treatments for cancers, and alkylating agents, such as TMZ, have been used clinically in chemotherapy as a single agent or in combination with PARP inhibitors for cancer treatments [6]. In addition, MMS and TMZ have been used to study genotoxicity by causing DNA damaging. These chemicals are known to cause, as part of their mechanism of action, DNA breaks that will trigger upregulations of PARP proteins for DNA damage signalling, thereby increasing the uptake of radiolabelled PARP inhibitors. Our *in vitro* studies and flow cytometry analyses significantly support the above-mentioned hypothesis, showing upregulation of γH2AX and PARP enzymes, indicating DNA damage and recruitment of proteins for DNA damage repair prior to the treatments of MMS and TMZ in AsPC1 and PSN1 cells, hence increasing cellular uptake of both rucaparib and olaparib. Of note, upregulated expression of ALDH2, a mitochondrial protein, was surprisingly observed prior to the DNA-damaging treatments, which is also believed contributing to the increased uptake of the PARP inhibitors in the treated cells. With this observation in mind, in-depth investigation will be needed to identify other protein or biomolecule that may contribute to the higher uptake of PARP inhibitors, prior to the DNA-damaging treatment, hence revealing more insights of DNA damage mechanism. This study shows the potential use of [^18^F]rucaparib or [^18^F]olaparib as PARP-targeting imaging agents to interrogate PARP expression levels during chemotherapy of cancer, aiding drug and radiation (bio-)dosimetry, treatment scheduling and gauging the emergence of resistance.

## Conclusion

Given the current challenges for PARP inhibitor cancer therapies, such as patient selection, treatment dosimetry and resistance to PARP therapy, the development of [^18^F]rucaparib and other radiolabelled PARP-targeting imaging agents may offer a way to overcome these challenges and improve treatment outcome for patients. The physicochemical properties of [^18^F]rucaparib, as an isotopologue of FDA-approved PARP inhibitor, rucaparib, may potentially allow real-time direct imaging of the whole body, and extraction of critical bio-information by interrogating PARP expressions, probing drug-target engagement and drug distribution and in-depth investigating off-binding effects, which may meet the current clinical needs in cancer imaging and therapy. Taken together, our study demonstrates the potentials of [^18^F]rucaparib as PARP imaging agent for cancer imaging.

## Supplementary Information

ESM 1 (DOCX 997 kb)
