# Supplementary material for: Imaging PARP with [18F]rucaparib in pancreatic cancer models
Source: Eur J Nucl Med Mol Imaging. 2022 May 26;49(11):3668–78. doi: 10.1007/s00259-022-05835-4 (PMC9399069; doi:10.1007/s00259-022-05835-4)
Supplement: Supplementary file 1 — (DOCX 997 kb) [file 259_2022_5835_MOESM1_ESM.docx]

**Supplemental information**

**Imaging PARP using [^18^F]rucaparib in pancreatic cancer models**

Chung Ying Chan*^a^*, Zijun Chen*^b^,* Gianluca Destro*^b^*, Mathew Veal*^a^*, Doreen Lau*^a^*, Edward O’Neill*^a^*, Gemma Dias*^a^*, Michael Mosley *^a^*, Veerle Kersemans^a^, Florian Guibbal*^b^*, Véronique Gouverneur*^b^* and Bart Cornelissen*^a,c*^*

*^a^* MRC Oxford Institute for Radiation Oncology, Department of Oncology, University of Oxford, Oxford, OX3 7DQ, UK.

*^b^* Department of Chemistry, Chemistry Research Laboratory, University of Oxford, 12 Mansfield Road, Oxford, OX1 3TA, UK.

*^c^* Department of Nuclear Medicine and Molecular Imaging, University Medical Center Groningen, University of Groningen, Groningen, The Netherlands.

**Supplemental Methods:**

Unless otherwise noted, all reagents were purchased from Sigma-Aldrich and used without further purification.

**Autoradiography**

PSN1 xenograft tissues were harvested and immediately flash-frozen using liquid nitrogen, and sectioned (10 µm) using a Leica CM1950 cryostat. Tissue sections were thaw-mounted onto Superfrost PLUS glass microscope slides (Menzel-Glaser, Thermo Scientific) and allowed to dry briefly at room temperature (RT). The slides were then fixed in 10% neutral buffered formalin overnight (NBF) for 10 min at RT, dried, and exposed to a storage phosphor screen (PerkinElmer, Super Resolution, 12.5 x 25.2 cm) in a standard X-ray cassette for 15 h at 4 °C. The phosphor screen was then imaged using a Cyclone® Plus Storage Phosphor System (PerkinElmer).

For tissues that were not used in autoradiography, selected tissues from mice were fixed in 10% NBF. Tissue was then flash frozen and stored at -80 °C overnight. Frozen tissue was sectioned (10 µm) using a Leica CM1950 cryostat. Tissue sections were thaw-mounted onto Superfrost PLUS glass microscope slides (Menzel-Glaser, Thermo Scientific). The slides were then stored at 4 °C until used.

**Immunohistochemical staining**

For immunohistochemical staining, slides were placed at RT for 5–10 mins to thaw. Slide incubation procedures were performed in glass Coplin type jars. The sections were dehydrated and rehydrated by sequential immersion in ethanol at 100% (twice), 70% and 50%, for 3 min at each concentration, with a final 1 min wash in water. The slides were then immersed in citrate buffer (10 mM sodium citrate pH 6.0) with 0.05% tween-20, and antigen retrieval was performed in an antigen-decloaking chamber (Biocare Medical) at 125 °C for 2.5 min, then 95 °C for 1 min. The slides were allowed to cool, then placed on ice.

The sections were processed using the EnVision FLEX DAB+ Substrate Chromogen System (Dako Omnis; Agilent Stockport UK). Tissue sections were blocked in Peroxidase and Alkaline Phosphatase Blocking Reagent (S2003) for 15 min at RT, then incubated overnight at 4 °C in tris-buffered saline buffer (TBS) with 1% bovine serum albumen (BSA) containing the following Atlas Antibodies (Sigma Aldrich, UK) anti-human PARP rabbit polyclonal antibodies at 1:150 dilution: anti-PARP1 antibody (HPA045168), anti-PARP2 antibody (HPA052003), and anti-PARP3 antibody (HPA067657). Following this incubation, the tissues were washed five times (5 min each) in phosphate-buffered saline (PBS), incubated for 30 min with Envision rabbit/mouse HRP polymer (K4065), and developed for 2.5 min using 1:50 dilution of DAB^+^ 2-component substrate (K3467). The sections were then washed in water twice (5 min each), and the nuclei counter-stained for 1 min in aqueous haematoxylin. This was followed by a 3 min wash in water, and four separate incubations in 100% ethanol (30 sec each), and two incubations in xylene (30 sec each). Coverslips were then mounted on DPX mounting media, and the slides left to set, and stored at 4 °C.

Positive PARP staining in the tissue sections appeared brown, and the nuclei were counter-stained pink by the haematoxylin. Section images were acquired by a brightfield Aperio (Leica biosystem ScanScope CS2, Milton Keynes UK), and image analysis performed using the Leica Aperio ImageScope software.

**Supplemental Table S1**: mRNA expression in AsPC1 and PSN1 cells. [www.ebi.ac.uk](http://www.ebi.ac.uk)

| Transcript Per Million | AsPC1 | PSN1 |
| --- | --- | --- |
| PARP1 | 109 | 229 |
| PARP2 | 22 | 44 |
| PARP3 | 30 | 27 |
| Tankyrase1 | 9 | 2 |
| Tankyrase2 | 40 | 61 |
| ALDH2 | 60 | 41 |
| IMPDH2 | 244 | 381 |

**Supplemental Table S2**: *Ex vivo* biodistribution of [^18^F]rucaparib (%ID/g) (0.87–11.38 MBq, A_m_ = 1.5–30.9 GBq/μmol) in PSN1 tumour-bearing mice at 1 h (n=3) and 2 h (n=6) post-injection time points.

| %ID/g | 1 h | | | 2 h | | | | | |
| --- | --- | --- | --- | --- | --- | --- | --- | --- | --- |
| Blood | 3.51 | 3.67 | 3.59 | 0.92 | 0.49 | 0.48 | 1.57 | 0.51 | 0.44 |
| Tumour | 5.25 | 6.05 | 5.16 | 2.58 | 2.10 | 1.74 | 2.60 | 3.45 | 2.99 |
| Heart | 13.27 | 16.24 | 17.47 | 2.44 | 2.15 | 1.73 | 1.73 | 1.61 | 1.27 |
| Lung | 17.76 | 25.71 | 27.51 | 8.77 | 6.24 | 4.58 | 9.27 | 5.27 | 8.24 |
| Liver | -- | 4.24 | 22.33 | 12.22 | 8.90 | 10.25 | 12.44 | 11.01 | 11.46 |
| Spleen | 32.86 | 41.87 | 47.79 | 32.75 | 20.87 | 15.30 | 23.45 | 16.14 | 14.89 |
| Stomach | 9.10 | 6.86 | 7.46 | 4.66 | 5.94 | 7.68 | 11.59 | 5.59 | 7.44 |
| Large intestine | 12.50 | 16.33 | 14.08 | 23.81 | 19.26 | 15.83 | 25.44 | 36.97 | 43.06 |
| Small intestine | -- | 33.79 | 46.24 | 16.44 | 9.50 | 19.42 | 22.39 | 10.51 | 13.44 |
| Pancreas | 19.47 | 23.54 | 23.22 | 13.99 | 9.45 | 9.78 | 11.07 | 9.35 | 7.70 |
| Kidney | 17.24 | 30.17 | 36.00 | 7.28 | 6.56 | 5.54 | 7.41 | 4.70 | 4.13 |
| Muscle | 2.63 | 4.25 | 3.13 | 2.51 | 1.58 | 1.90 | 2.77 | 1.57 | 1.57 |
| Fat | 6.53 | 4.98 | 6.82 | 1.32 | 0.74 | 0.84 | 1.78 | 1.03 | 0.86 |
| Bone | 4.90 | 6.79 | 5.26 | 3.49 | 2.71 | 2.38 | 3.10 | 2.18 | 2.26 |

**Supplemental Table S3**: *Ex vivo* biodistribution of [^18^F]rucaparib (%ID/g) (0.87–2.47 MBq, A_m_ = 5.5 GBq/μmol) in PSN1 tumour-bearing mice (n=3) with or without the co-injection of olaparib or rucaparib (0.5 mg) as blocking agent.

| %ID/g | Non-treated | | | Olaparib-blocked | | | Rucaparib-blocked | | |
| --- | --- | --- | --- | --- | --- | --- | --- | --- | --- |
| Blood | 0.92 | 0.49 | 0.48 | 0.62 | 0.49 | 0.38 | 0.04 | 0.29 | 0.36 |
| Tumour | 2.58 | 2.10 | 1.74 | 2.83 | 1.36 | 1.23 | 0.11 | 1.20 | 1.12 |
| Heart | 2.44 | 2.15 | 1.73 | 1.07 | 0.82 | 0.83 | 0.06 | 0.76 | 0.51 |
| Lung | 8.77 | 6.24 | 4.58 | 5.83 | 5.49 | 6.59 | 0.35 | 4.77 | 2.50 |
| Liver | 12.22 | 8.90 | 10.25 | 13.15 | 9.74 | 9.86 | 0.62 | 5.66 | 7.42 |
| Spleen | 32.75 | 20.87 | 15.30 | 3.05 | 3.03 | 2.63 | 0.19 | 1.85 | 1.85 |
| Stomach | 4.66 | 5.94 | 7.68 | 12.09 | 9.73 | 6.47 | 0.53 | 6.53 | 4.49 |
| Large intestine | 23.81 | 19.26 | 15.83 | 33.55 | 35.89 | 30.25 | 3.24 | 45.68 | 25.75 |
| Small intestine | 16.44 | 9.50 | 19.42 | 8.33 | 9.66 | 6.32 | 0.56 | 11.02 | 5.09 |
| Pancreas | 13.99 | 9.45 | 9.78 | 4.67 | 4.17 | 4.43 | 0.31 | 3.02 | 2.76 |
| Kidney | 7.28 | 6.56 | 5.54 | 5.09 | 3.73 | 3.29 | 0.24 | 2.32 | 2.51 |
| Muscle | 2.51 | 1.58 | 1.90 | 1.19 | 1.07 | 1.10 | 0.10 | 1.36 | 0.74 |
| Fat | 1.32 | 0.74 | 0.84 | 0.73 | 0.74 | 0.75 | 0.04 | 0.41 | 0.31 |
| Bone | 3.49 | 2.71 | 2.38 | 1.34 | 1.17 | 1.13 | 0.10 | 1.21 | 0.90 |
| Brain | 0.09 | 0.06 | 0.06 | 0.06 | 0.06 | 0.04 | 0.00 | 0.06 | 0.06 |

**Supplemental data:**

**Supplemental Figure S1:** Mean fluorescence intensity (MFI) for the staining of PARP1, 2 and 3, Tankyrase 1/2, ALDH2 and IMPDH2 enzymes in AsPC1 and PSN1 cells assessed by flow cytometry analysis.


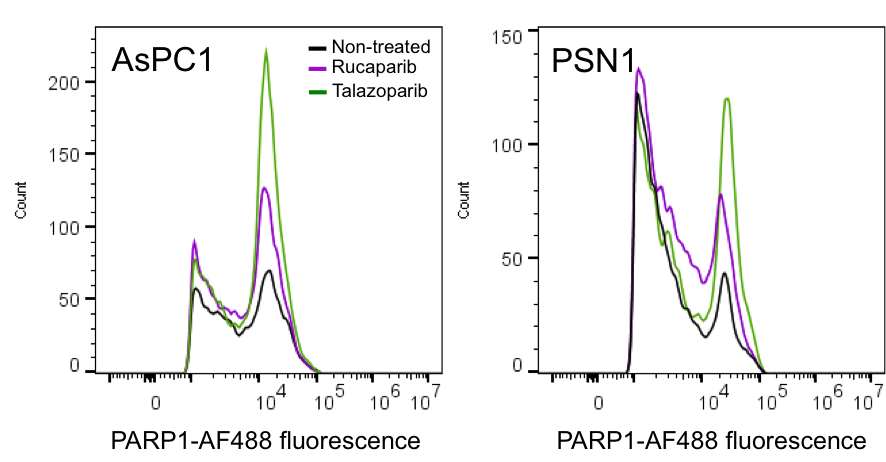


**Supplemental Figure S2**: Representative frequency histograms probing for PARP1 expressions in AsPC1 and PSN1 cells after exposed to PARP inhibitors (10 μM) for 3 h.

**Supplemental Figure S3**: Uptake and retention (at 3 h) of [^Total^F]olaparib (300 nM, [^18^F]olaparib: 400 kBq, 8.5 GBq/μmol) in cells (AsPC1 and PSN1) treated with DNA damaging reagents (MMS and TMZ). Asterisks indicate levels of significance: *, *P* < 0.05; **, *P* < 0.01.

**Supplemental Figure S4**: Cell retention of [^Total^F]olaparib (left, 75.6 μM, molar activity: 2.8 GBq/μmol) and [^Total^F]rucaparib (right, 77.4 μM, molar activity: 4.8 GBq/μmol) in AsPC1 and PSN1 cells.

**
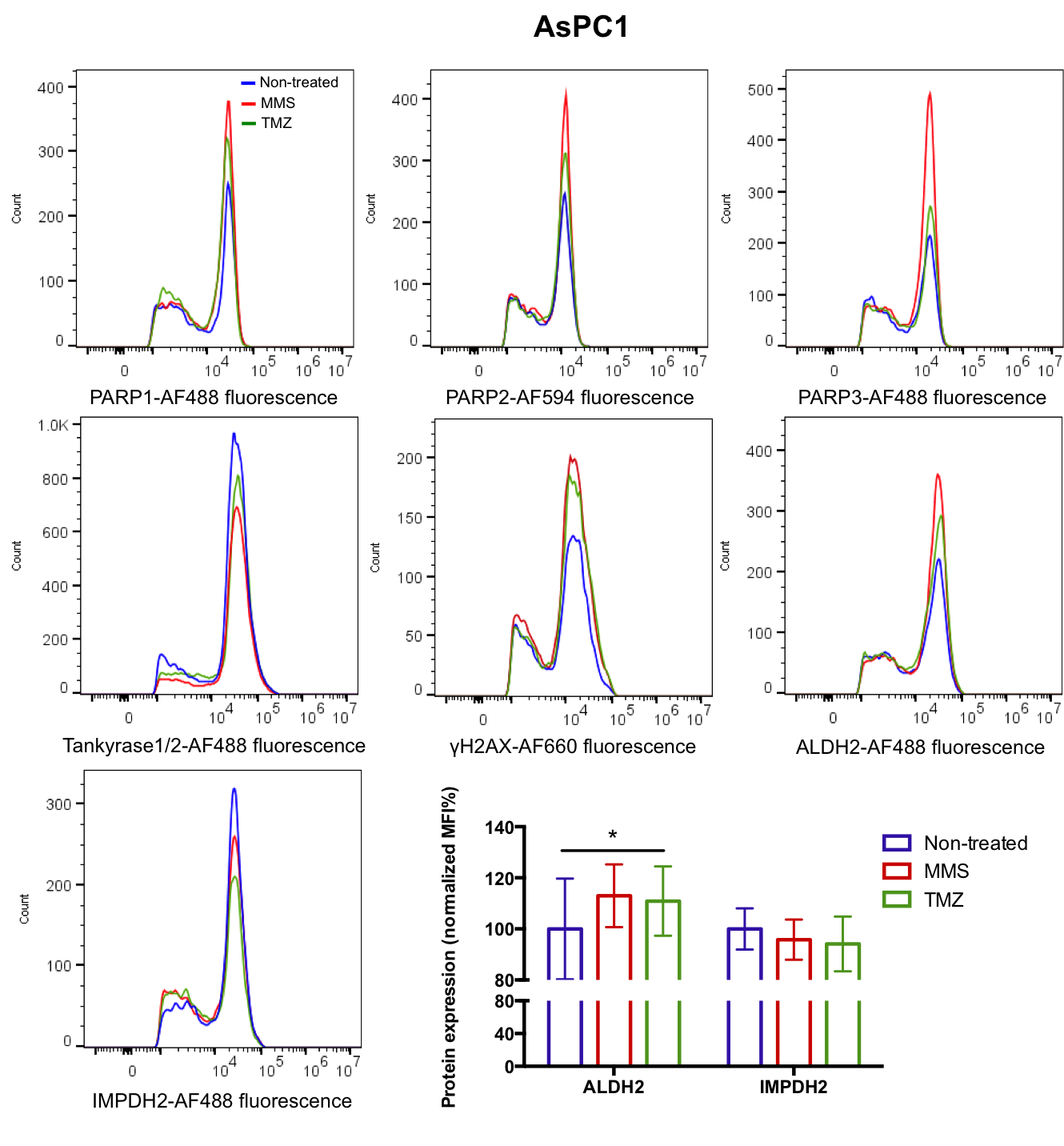
**

**Supplemental Figure S5**: Representative frequency histograms probing for protein expressions in AsPC1 cells after exposed to MMS or TMZ (100 μM) for 3 h, and mean fluorescence intensity (MFI) for ALDH2 and IMPDH2 assessed by flow cytometry analysis. Asterisks indicate levels of significance: *, *P* < 0.05.


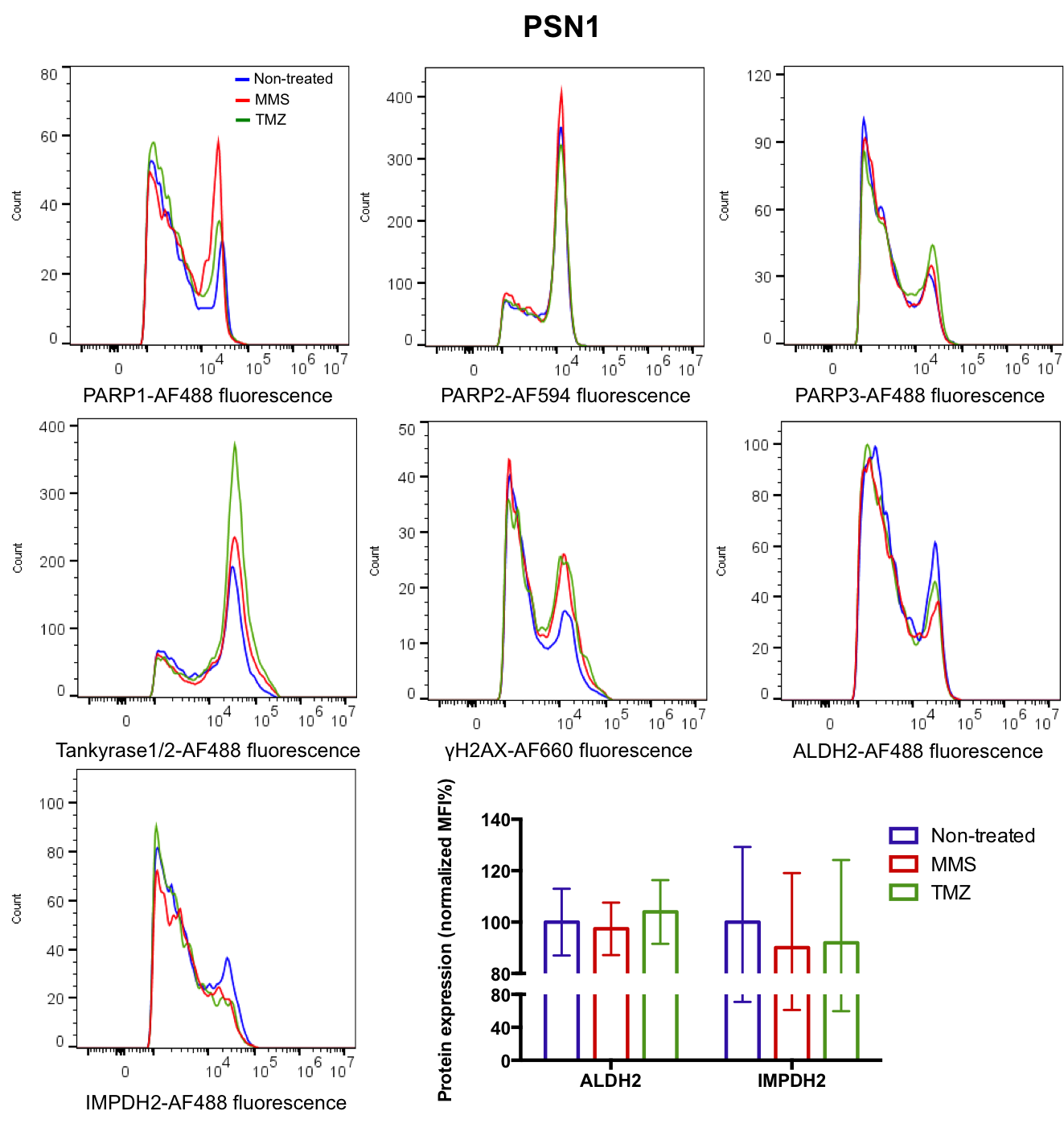


**Supplemental Figure S6**: Representative frequency histograms probing for protein expressions in PSN1 cells after exposed to MMS or TMZ (100 μM) for 3 h, and mean fluorescence intensity (MFI) for ALDH2 and IMPDH2 assessed by flow cytometry analysis.

**Supplemental Figure S7**: [^18^F]rucaparib clearance in blood and liver of PNS1 tumour-bearing mice (n=3).
